# Self-Esteem and Problematic Drinking in China: A Mediated Model

**DOI:** 10.1371/journal.pone.0140183

**Published:** 2015-10-09

**Authors:** Hui Zhai, Yanjie Yang, Hong Sui, Wenbo Wang, Lu Chen, Xiaohui Qiu, Xiuxian Yang, Zhengxue Qiao, Lin Wang, Xiongzhao Zhu, Jiarun Yang

**Affiliations:** 1 Department of Medical Psychology, Public Health Institute of Harbin Medical University, Harbin, China; 2 Department of Epidemiology and Health Statistics, Public Health Institute of Harbin Medical University, Harbin, China; 3 Department of Endocrinology, Peking Union Medical College Hospital, Beijing, China; 4 Department of Medical Psychology, Second Xiangya Hospital, Central South University, Changsha, China; Harvard Medical School, UNITED STATES

## Abstract

**Background:**

Although self-esteem is related to problematic drinking, the mechanisms by which it affects drinking remain unclear. The purpose of this study was to determine whether coping mechanisms mediate the relationship between self-esteem and problematic drinking among Chinese men and women with alcohol use disorders and to recommend appropriate interventions for drinking problems.

**Methods:**

A cross-sectional survey was conducted in Harbin, Heilongjiang Province, China. A sample of 5,689 community residents was screened, and 517 male and 172 female problematic drinkers were chosen to participate in this study. A self-esteem scale, a coping questionnaire and an alcohol use disorder identification test were completed in order to assess participants’ self-esteem, coping mechanisms and alcohol use disorders, respectively. Participants’ socio-demographic data were also gathered at this stage. The resulting data were examined via descriptive statistics, correlations and bootstrap analyses.

**Results:**

Lower self-esteem levels were related to problematic drinking, and there were no gender differences in the relationship between self-esteem and problematic drinking. A relationship between low self-esteem and negative coping was observed only in men. Negative coping thus mediated the relationship between self-esteem and problematic drinking among men, but this was not the case for women. Positive coping did not mediate the relationship between self-esteem and problematic drinking among the participants, regardless of gender.

**Conclusions:**

Self-esteem and coping strategies are correlated among problematic drinkers. In addition, there are gender differences in the manners in which negative coping mediates the relationship between self-esteem and problematic drinking. Problematic drinking interventions directed at males should simultaneously address low self-esteem and negative coping.

## Introduction

Alcohol use disorders are a major public health issue and a leading cause of death worldwide; they account for 3.2% of deaths (1.8 million people) per year. Of these deaths, 80% occur in developing countries [1]. In China, a developing country, nearly three-quarters of the population (90% of men and 55% of women) consumes alcohol. Moreover, the prevalence of alcohol consumption is growing in China among both men and women [2]. On average, male drinkers consumed 47.8 g of alcohol per drinking day, whereas females consumed 19.1 g. Among the current drinkers, proportions of excessive drinking, frequent dinking and binge drinking were 62.7%, 26.3%, 57.3% for men and 51.0%, 7.8%, 26.6% for women, respectively [3]. The increasing prevalence of alcohol disorders has harmful effects on individuals, families and societies [4]. Although the percentage of people engaging in problematic alcohol use in China has rapidly risen and the consequences of it have become increasingly severe for both genders, the factors that influence it and the gender disparities related to it in China have remained unclear. This paper attempts to address these gaps in the literature.

People in China are known to engage in self-suppression to maintain harmony [5]. Their speech and behavior are always controlled in order to maintain a cooperative environment, and they often inhibit or change their own desires to adapt to those of others. This emphasis on the collective is rooted in Chinese culture and tends to devalue individual instincts. China is also an intensely competitive country with a large population; thus, its people are under considerable pressure to work in fiercely competitive conditions. Chinese people frequently review their personal weaknesses and excessively focus on the merits of others. Hence, the self-awareness of the Chinese people differs from that of foreigners, and Chinese people tend to exhibit lower levels of self-esteem. Because there is a correlation between low self-esteem and alcohol problems, alcohol problems may be more common and severe among Chinese people.

Having low self-esteem often increases the likelihood that individuals will use alcohol to manage increases in negative feelings [6]. Individuals with low self-esteem are typically characterized as vulnerable, anxious, lonely and depressed [7]. These individuals may also experience higher levels of stress and lower levels of self-esteem as an indirect result of using alcohol to manage their stress levels [8]. Thus, self-esteem levels typically serve as a predictor of alcohol problems. However, inconsistent results regarding the relationship between self-esteem and problematic drinking have been obtained. Some studies have found that individuals with high self-esteem were prone to using alcohol [9]. The consistency theory, which states that individuals with high self-esteem engage in risky behaviors to maintain a consistent self-concept and to diminish conflicting information, can be used to explain this finding [10]. Other studies have observed no association between self-esteem and the severity of alcohol use [11]. Thus, it is necessary to examine and present conclusive findings regarding the relationship between self-esteem and excessive alcohol use.

Although self-esteem and alcohol problems frequently occur together, alcohol problems may not necessarily be a consequence of self-esteem levels. Coping may serve as a mediator between self-esteem and alcohol consumption. D’ Zurilla found that low self-esteem can promote anger and hostility, which can cause individuals to address problems in an unhealthy manner and to employ impulsive and escapist defense mechanisms [12]. Individuals consume alcohol to manage negative emotions because they lack effective coping mechanisms. Unhealthy coping mechanisms increase the reliance on alcohol, and in turn, excessive drinking limits the capacity to engage in adaptive coping behaviors [13]. David Dunkley defines coping strategies as the thoughts and behaviors that people use to manage the demands of situations that elicit negative emotions [14]. People develop a variety of coping strategies to manage various emotions [13] in their lives and establish their own personal drinking patterns [15]. Individuals who engage in negative coping strategies may experience more negative emotions and problems over time [16]. Thus, negative coping is frequently associated with increased drinking related to negative emotions [17]. In contrast, positive coping strategies, which focus on processing or resolving situations, often serve as protective strategies that prevent drinking problems [18, 19]. Previous studies have demonstrated that maladaptive coping strategies can lead to drinking problems [20, 21] and that alcohol consumption may be treated through improved coping strategies that allow individuals to adapt to adverse environments [22]. However, some studies of adults have indicated that, in some cases, coping mechanisms may not regulate the relationship between negative emotions and drinking [23]. Furthermore, in other studies, the role of negative coping in managing negative emotions and alcohol consumption was observed only in men [24]. Thus, there may be gender differences in the mediating effects of coping mechanisms on the relationship between self-esteem and alcohol use problems; studies must be pursued in this area.

Previous studies have neglected to account for the mediating effects of self-esteem on alcohol consumption, which has led to conflicting conclusions regarding the effects of self-esteem and coping on problematic drinking. The current study examines the relationships between self-esteem, coping mechanisms and problematic drinking, and it specifically investigates the effects of coping mechanisms on self-esteem and problematic drinking. Thus, our goal is to present comprehensive findings on the relationships between self-esteem, coping mechanisms and problematic drinking. Analytic models are tested in a sample of individuals with drinking problems. We hypothesize that self-esteem levels are related to problematic drinking and that the mediating effects of coping differ in males and females.

## Materials and Methods

### Participants

The research described in this paper meets the ethical guidelines of the ethics committee of Harbin Medical University. Approval for this study was granted by the Ethics Committee of Harbin Medical University. The nature and purposes of the study were explained to all of the participants. Participation was completely voluntary, with no economic or other motivations involved, and each participant signed a written statement of informed consent. The sample was drawn from the Ministry of National Science and Technology plan survey, which is a large community survey of health and psychological health information awareness. A cross-sectional study was conducted using residents of Harbin, which is a city in northeastern China. A multi-stage randomized cluster sampling method was employed to select the sample. In the first stage, after considering the operational and scientific aspects of the study, we selected two roads (Nangang and Dacheng) using a simple random sampling method. Then, we employed a probability proportionate to size sampling method to select four neighborhoods located along each road. In each neighborhood, we selected 5 residential buildings. The participants were local residents of the area or had lived in the selected buildings for more than half a year and were at least 15 years of age. The researchers entered the respondents’ homes to conduct the face-to-face surveys. Before completing the questionnaires, all of the participants provided their consent to participate in the survey. The researchers read the survey questions aloud to respondents who could not read and answered any questions that respondents had regarding unclear questions. The questionnaires were returned to the researchers immediately after they were completed. Interviewees who were not home during the first visit were visited two additional times. Individuals who were not home on three occasions or who did not wish to participate were excluded from the study. Although we initially planned to invite 5,000 community residents to participate in the survey, we ultimately invited 5,689 individuals to account for refusals.

### Measures

We collected sociodemographic information from the participants regarding their gender, age, marital status, educational background and household income.

As part of the survey, a translated version of the Rosenberg Self-Esteem Scale (RSE) was administered in order to assess each participant’s self-esteem [25]. It included 10 items that were rated on the following scale: strongly disagree, disagree, agree and strongly agree. Each question tested self-satisfaction agreement levels, and the total score was the sum of all of the items. Higher total scores indicated higher levels of self-esteem. All of the items exhibited good reliability and validity.

Coping styles were assessed using the Simplified Coping Style Questionnaire, which is based on the Ways of Coping Questionnaire (WCQ) by Folkman and Lararus [26, 27]. Because of differences in cultural backgrounds, foreign scales are not well-adapted to the population in China. Additionally, coping styles have a variety of patterns, but all of the coping styles seem to display the same characteristics. Some styles contain a more positive composition; these styles include methods such as support-seeking and changes to value systems. Others are based on a more negative composition, with mechanisms such as avoidance and outlets. A simplified coping style questionnaire is created by mixing Chinese characteristics and actual applications of coping styles. The 20-item scale includes two subscales: one positive and one negative. Items 1–12 form the positive coping subscale, and items 13–20 form the negative coping subscale. The answer choices include never, occasionally, sometimes and frequently, which give scores of 0, 1, 2, and 3, respectively. Higher scores indicate a greater likelihood that positive or negative coping mechanisms are being employed. The Cronbach’s alpha values were 0.90 for the questionnaire as a whole and were 0.89 and 0.78 for the positive and negative coping subscales, respectively. The coping questionnaire exhibited good validity with respect to differences in age, gender, occupation and ethnicity.

We used the Alcohol Use Disorders Identification Test questionnaire (AUDIT) to identify respondents who had drinking problems [28]. Of the 10 items on this questionnaire, 3 measure the amount and frequency of alcohol consumption, 3 measure alcohol dependence and four measure problems related to alcohol consumption. Scores can range from 0 to 40, with scores ≥8 indicating problematic drinking. A higher one-dimensional total score indicates a more severe alcohol problem. The AUDIT can accurately identify problems associated with alcohol regardless of each respondent’s educational background, age or gender.

### Statistical Analysis

Analyses were conducted to determine the mediating effect of coping mechanisms on the relationship between self-esteem and problematic drinking. We used SPSS 18.0 and SPSS Bootstrapping to analyze the data using the following procedure.

First, marital status, educational level and household income distributions were determined via descriptive statistics. Second, we analyzed the associations among age, self-esteem, negative coping, positive coping and problematic drinking using correlation analyses. We then tested whether self-esteem predicted problematic drinking (path c) and coping strategies (path a). For path b, coping strategies were added to the regression model that controlled for sociodemographic variables (age, marital status, educational level and household income) in order to determine the relationships between various coping mechanisms and problematic drinking. With path c’, we determined the association between self-esteem and problematic drinking when controlling for age, marital status, educational level and household income. The main effects of paths a, b, c and c’ were determined based on four criteria. After all four criteria for testing mediation were fulfilled, we calculated the (a*b)/c value to identify the mediating roles of the different coping strategies. When all of the pathways suggested mediation, SPSS Bootstrapping was employed. In this study, 5,000 bootstrap samples were used.

## Results

### Sample characteristics

A total of 5,689 community residents were investigated, and we ultimately collected 5,466 valid and complete questionnaires. Of these participants, 689 participants (517 males and 172 females) had alcohol use disorders. The average age of the respondents with drinking problems was 39.15±12.09 years; the average age of the males was 39.78±11.57 years, with a range of 15 to 77 years, and the average age of the females was 37.27±13.40 years, with a range of 16 to 71 years. Differences between males and females with respect to marital status, educational levels and household incomes were significant (χ^2^ = 5.51, P<0.05; χ^2^ = 6.41, P<0.05 and χ^2^ = 5.55, P = 0.14, respectively); these results are displayed in Table 1. The mean and standard deviation values for ages, coping mechanisms, self-esteem levels and problematic drinking according to marital statuses, educational levels and household incomes are presented in Table 2.

**Table 1:** Predictor variables among males and females in China.

Variables	Male (N = 517)	Female (N = 172)	*χ* ^2^	P
	Mean(%)	Mean(%)		
**Marital status**			5.51	0.02
Single	127 (24.6%)	58 (33.7%)		
Married	390 (75.4%)	114 (66.3%)		
**Educational level**			6.41	0.04
Junior or lower	246 (47.6%)	101 (58.7%)		
College	251 (48.5%)	66 (38.4%)		
Graduate or higher	20 (3.9%)	5 (2.9%)		
**Household income**			5.55	0.14
0–999	61 (11.8%)	23 (13.4%)		
1000–1999	155 (30.0%)	66 (38.4%)		
2000–2999	159 (30.8%)	46 (26.7%)		
3000-	142 (27.5%)	37 (21.5%)		

**Table 2:** Age, marital status, education level characteristic and means and standard deviations of variables.

	Males						Females					
	N	Age	Self-esteem	Negative coping	Positive coping	Problematic drinking	N	Age	Self-esteem	Negative coping	Positive coping	Problematic drinking
		Mean(SD)	Mean(SD)	Mean(SD)	Mean(SD)	Mean(SD)		Mean(SD)	Mean(SD)	Mean(SD)	Mean(SD)	Mean(SD)
Total	517	39.78(11.57)	29.50 (4.00)	9.95 (4.96)	21.84(6.54)	13.95 (5.32)	172	37.27(13.40)	28.18(3.92)	11.32(4.68)	20.74(6.49)	15.28(6.86)
Marital status												
Single	127	30.27(11.48)	28.93(3.79)	10.39(4.78)	20.69(6.53)	14.58(5.97)	58	30.12(13.95)	28.00(3.71)	10.81(4.91)	20.21(6.58)	15.98(6.87)
Married	390	42.87(9.79)	29.69(4.06)	9.81(5.02)	22.21(6.52)	13.74(5.08)	114	40.91(11.58)	28.27(4.03)	11.58(4.55)	21.02(6.46)	14.93(6.86)
Educational level												
Junior or lower	246	41.93(12.37)	28.74(3.90)	10.60(4.93)	21.22(6.41)	14.05(5.53)	101	40.72(14.24)	28.32(3.98)	11.94(4.64)	20.66(6.70)	16.14(7.05)
College	251	37.93(10.68)	30.16(3.98)	9.41(4.93)	22.43(6.63)	13.83(5.15)	66	32.21(10.67)	27.76(3.72)	10.56(4.69)	20.73(6.20)	14.38(6.54)
Graduate or higher	20	36.50(6.93)	30.70(4.00)	8.70(5.03)	22.00(6.73)	14.05(4.96)	5	34.40(5.03)	31.00(4.64)	8.80(3.49)	22.60(7.16)	10.00(2.35)
Household income												
0–999	61	40.38(11.64)	27.66(3.97)	10.77(5.39)	21.34(6.62)	13.77(5.69)	23	36.35(13.72)	26.91(4.01)	13.83(4.75)	21.83(7.46)	19.04(8.41)
1000–1999	155	38.88(12.07)	29.68(4.03)	10.00(4.92)	21.54(6.79)	14.39(5.51)	66	36.21(14.10)	28.35(3.87)	11.08(4.02)	19.92(5.92)	15.11(5.68)
2000–2999	159	40.68(11.72)	30.12(3.75)	9.79(4.91)	22.26(6.37)	13.64(4.83)	46	38.41(12.82)	27.93(3.32)	11.43(5.18)	20.46(6.34)	14.04(6.07)
3000-	142	39.49(10.83)	29.42(4.08)	9.73(4.91)	21.90(6.46)	13.89(5.48)	37	38.32(12.99)	28.97(4.53)	10.05(4.64)	21.89(7.03)	14.81(8.04)

### Correlations between age, coping, self-esteem and problematic drinking

Correlations between age, self-esteem, coping and problematic drinking according to gender are displayed in Tables 3 and 4. The relationships between self-esteem and coping mechanisms, self-esteem and problematic drinking, and negative coping and problematic drinking were significant for both males and females. Two of these associations were negative: self-esteem and negative coping and self-esteem and problematic drinking. Positive coping was positively associated with problematic drinking for male respondents but not for female respondents. No significant relationships were observed between age and any other variables.

**Table 3:** Correlations between age, negative coping, positive coping, self-esteem and problematic drinking among males.

Variables	1	2	3	4	5
1. Age	**——**				
2. Self-esteem	0.008	**——**			
3. Negative coping	0.054	-0.275^a^	**——**		
4. Positive coping	0.047	0.379^a^	0.204^a^	**——**	
5. Problematic drinking	-0.050	-0.212^a^	0.189^a^	-0.159^a^	**——**

^a^ P<0.01

**Table 4:** Correlations between age, avoidant coping, approach coping, self-esteem and problematic drinking among females.

Variables	1	2	3	4	5
1. Age	**——**				
2. Self-esteem	0.008	**——**			
3. Negative coping	0.054	-0.275^a^	**——**		
4. Positive coping	0.047	0.379^a^	0.204^a^	**——**	
5. Problematic drinking	-0.050	-0.212^a^	0.189^a^	-0.159^a^	**——**

^a^ P<0.01

### Coping as a mediator of the relationship between self-esteem and problematic drinking

Tables 5 and 6 present the path coefficients, a*b products and BCa 95% CI values when controlling for age, marital status, educational level and household income in the model. First, self-esteem was negatively associated with problematic drinking for both males and females (path c) (c = -0.312, p<0.01 for males; c = -0.513, p<0.01 for females). Second, self-esteem was associated with both negative and positive coping mechanisms (path a) (negative coping: a = -0.313, p<0.01 for males; a = -0.310, p<0.01 for females; positive coping: a = 0.578, p<0.01 for males; a = 0.311, p<0.05 for females). A negative association was found between self-esteem and negative coping, and a positive association was found between self-esteem and positive coping. Third, negative coping was positively associated with problematic drinking among males (path b) after controlling for age, marital status, educational level and household income (b = -0.180, p<0.01). No relationships were observed between positive or negative coping and problematic drinking among the female respondents. Thus, we conclude that negative coping mediated the relationship between self-esteem and problematic drinking among the male respondents but not the female respondents. Finally, positive coping did not mediate the relationship between self-esteem and problematic drinking among any of the respondents, regardless of gender.

**Table 5:** Regression analysis results, with problematic drinking as outcome and negative coping and positive coping as mediators among males.

Predictors	Path coefficients				a* b^e^ (BCa 95%CI)^f^
	c^a^	a^b^	b^c^	c’^d^	
Negative coping	-0.312^g^	-0.314^g^	0.182^g^	-0.255^g^	-0.057(-0.104, -0.027)
Positive coping	-0.312^g^	0.578^g^	-0.057	-0.280^g^	-0.033(-0.077, -0.008)

^a^ associations of self-esteem with problematic drinking

^b^ associations of self-esteem with negative coping/ positive coping

^c^ associations between negative coping/ positive coping and problematic drinking after controlling for the predictor variables [Age, marital status, educational levels and household income]

^d^ associations of self-esteem with problematic drinking after adding negative coping/ positive coping as mediator

^e^ the product of a and b;

^f^ the bias-corrected and accelerated 95% confidence interval;

^g^ p<0.01

**Table 6:** Regression analysis results, with problematic drinking as outcome and negative coping and positive coping as mediators among females.

Predictors	Path coefficients				a* b^e^ (BCa 95%CI)^f^
	c^a^	a^b^	b^c^	c’^d^	
Negative coping	-0.513^h^	-0.317^h^	-0.066	-0.523^h^	0.021 (-0.055, 0.115)
Positive coping	-0.513^h^	0.308^g^	-0.003	-0.501^h^	-0.001 (-0.053, 0.050)

^a^ associations of self-esteem with problematic drinking

^b^ associations of self-esteem with negative coping/ positive coping

^c^ associations between negative coping/ positive coping and problematic drinking after controlling for the predictor variables [Age, marital status, educational levels and household income]

^d^ associations of self-esteem with problematic drinking after adding negative coping/ positive coping as mediator

^e^ the product of a and b;

^f^ the bias-corrected and accelerated 95% confidence interval;

^g^ p<0.05

^h^ p<0.01

The direct pathway between self-esteem and problematic drinking (path c’) was significant for the male respondents when negative coping mediated the model (c’ = -0.257, p<0.01), which indicates that negative coping served as a partial mediator of the relationship between self-esteem and problematic drinking among the male respondents. Therefore, we tested the total effect of the independent variable on the dependent variable for path c and employed the (a*b)/c formula to calculate the effect size of the mediation. For the male respondents, the negative coping mediation effects for the self-esteem to problematic drinking path were found to be 18.32%.

## Discussion

Self-esteem is considered a predictive variable for problematic drinking [29], and negative and positive coping mechanisms are often cited as having opposing effects on it [18]. This study of self-esteem and coping mechanisms has revealed that negative coping mediates the relationship between self-esteem and problematic drinking and that this relationship varies with gender. For males, there is a mediation effect; self-esteem affects problematic drinking via negative coping, and self-esteem directly affects problematic drinking. For females, negative coping did not mediate the relationship between self-esteem and problematic drinking. Finally, positive coping did not mediate the relationship between self-esteem and problematic drinking for respondents of either gender.

Previous studies have demonstrated that self-esteem is associated with alcohol use problems [6,9,11]. However, results from these studies regarding whether self-esteem levels are affected by irregular alcohol use and whether self-esteem levels affect problematic drinking among people in China have been inconclusive. First, all of our significant findings on the relationship between self-esteem and problematic drinking indicate that lower self-esteem is related to problematic drinking. This finding is consistent with the finding of Tomaka and his colleagues that individuals with low self-esteem often abuse alcohol when confronted with stress [30]. Second, the present study suggests that Chinese individuals with low self-esteem report experiencing more alcohol- related problems. This finding fills the gap in this neglected research area regarding the effect of self-esteem on problematic drinking in China.

We found associations between self-esteem and negative and positive coping mechanisms. Among problematic drinkers, those with lower levels of self-esteem frequently engaged in negative coping, whereas those with higher levels of self-esteem typically utilized positive coping. This pattern is consistent with Stein and his colleagues’ finding that lower levels of self-esteem are positively associated with negative coping strategies and negatively associated with positive coping strategies [31]. People who feel devalued often suffer from limited social support. Access to social support can increase the use of positive coping methods by increasing an individual’s self-esteem [12]. Furthermore, self-esteem levels regulate alcoholics’ moods. This affects their coping methods [12] and suggests that—in addition to enhancing social support—increased self-esteem can help discourage negative coping and enhance and facilitate positive coping among alcoholics.

The nature of the associations between self-esteem, coping mechanisms and problematic drinking suggest that negative coping only mediates the relationship between self-esteem and problematic drinking in men. It appears that when men with lower levels of self-esteem adopt negative coping mechanisms, they may be more likely to consume alcohol. According to the self-discrepancy theory, the discrepancy between the actual and ideal self is uniquely presented via personal self-esteem. This discrepancy often promotes the development of dejection-related emotions [32]. Men with high drinking expectancies are more likely than women with high drinking expectancies to report coping with negative feelings by abusing alcohol [33]. When individuals experiencing negative emotions utilize negative coping mechanisms, problematic drinking is common among those with high drinking expectancies; however, it is uncommon among those with low drinking expectancies [24]. Thus, men with lower levels of self-esteem may be more prone to utilizing negative coping mechanisms and thus present with more drinking problems. In addition, positive coping did not act as a mediator between self-esteem and problematic drinking for any of the participants in this study, regardless of their gender. Positive coping appears to have no mediating effect on problematic drinking in individuals with low self-esteem. Thus, self-esteem and negative coping play important roles in alcohol use problems, and further studies should be performed to investigate the role of positive coping in problematic drinking.

Despite these significant findings, the current study also has a number of limitations. First, the study involved a cross-sectional investigation, which meant that we could not determine the directions of causality among self-esteem, negative coping, positive coping and problematic drinking; however, our results are consistent with our hypotheses and are based on firm theoretical foundations. Second, we only studied residents of Harbin, despite being interested in problematic drinking in China as a whole. Regional differences in China should be considered in future studies. We plan to examine this issue further by studying problematic drinking in other regions and by conducting a cohort study.

In summary, self-esteem and coping strategies not only have correlations with each other but also have independent associations with problematic drinking. People with drinking problems often have low self-esteem, are more prone to using negative coping and are more reluctant to use positive coping. In men, low self-esteem and negative coping play important roles at the same time, and negative coping can mediate the association between low self-esteem and problematic drinking; this finding was not observed in women. Psychotherapy focused on self-esteem and coping strategies can benefit problematic drinkers and improve clinical results. People, and men in particular, who have low self-esteem and rely on negative coping should alert alcohol treatment professionals to the possibility of problematic drinking.

Clinicians should pay attention to self-esteem and coping strategies when working with individuals who are facing negative life events and indulging in poor mood and should actively utilize proper interventions. This could include using the “deficit model” to teach individuals skills that can improve self-esteem [34, 35] and cognitive behavioral training to reduce the use of negative coping and increase the use of positive coping [36]. When working with male drinkers, attention should especially be paid to two factors that mediate self-esteem: negative coping and problematic drinking. The use of single interventions for self-esteem and negative coping, especially for male drinkers, is not enough. Clinicians should develop a more comprehensive and effective treatment plan to improve self-esteem and reduce negative coping among people with alcohol use problems.
